# Systematic symptom and problem assessment at admission to the palliative care ward – perspectives and prognostic impacts

**DOI:** 10.1186/s12904-020-00576-3

**Published:** 2020-05-28

**Authors:** Anja Coym, Anneke Ullrich, Lisa Kathrin Hackspiel, Mareike Ahrenholz, Carsten Bokemeyer, Karin Oechsle

**Affiliations:** Department of Oncology, Hematology and BMT, Palliative Care Unit, University Medical Center Hamburg-Eppendorf, Martinistr. 52, 20246 Hamburg, Germany

**Keywords:** Symptom assessment, Palliative care, Self-assessment, Patient-reported outcome, Physical, Psychosocial

## Abstract

**Background:**

Symptom assessment is essential in palliative care, but holds challenges concerning implementation and relevance. This study aims to evaluate patients’ main symptoms and problems at admission to a specialist inpatient palliative care (SIPC) ward using physician proxy- and patient self-assessment, and aims to identify their prognostic impact as well as the agreement between both assessments.

**Methods:**

Within 12 h after admission, palliative care specialists completed the Symptom and Problem Checklist of the German Hospice and Palliative Care Evaluation (HOPE-SP-CL). Patients either used the new version of the minimal documentation system for patients in palliative care (MIDOS) or the Integrated Palliative Care Outcome Scale (IPOS) plus the Distress Thermometer (DT).

**Results:**

Between 01.01.2016–30.09.2018, 1206 patients were included (HOPE-SP-CL 98%; MIDOS 21%, IPOS 34%, DT 27%) whereof 59% died on the ward. Proxy-assessment showed a mean HOPE-SP-CL Total Score of 24.6 ± 5.9 of 45. Most frequent symptoms/problems of at least moderate intensity were weakness (95%), needs of assistance with activities of daily living (88%), overburdening of family caregivers (83%), and tiredness (75%). Factor analysis identified four symptom clusters (SCs): (1) Deteriorated Physical Condition/Decompensation of Home Care, (2) Emotional Problems, (3) Gastrointestinal Symptoms and (4) Other Symptoms. Self-assessment showed a mean MIDOS Total Score of 11.3 ± 5.3 of 30, a mean IPOS Total Score of 32.0 ± 9.0 of 68, and a mean distress of 6.6 ± 2.5 of 10. Agreement of self- and proxy-assessment was moderate for pain (ƙ = 0.438) and dyspnea (ƙ = 0.503), fair for other physical (ƙ = 0.297 to 0.394) and poor for psychological symptoms (ƙ = 0.101 to 0.202). Multivariate regression analyses for single symptoms and SCs revealed that predictors for dying on the SIPC ward included impaired ECOG performance status, moderate/severe dyspnea, appetite loss, tiredness, disorientation/confusion, and the SC Deteriorated Physical Condition/Decompensation of Home Care.

**Conclusion:**

Admissions to a SIPC ward are mainly caused by problems impairing mobility and autonomy. Results demonstrate that implementation of self- and reliability of proxy- and self-assessment is challenging, especially concerning non-physical symptoms/problems. We identified, specific symptoms and problems that might provide information needed for treatment discussions regarding the medical prognosis.

## Background

Systematic symptom assessment by palliative care specialists has been established as a routine measure within specialist inpatient palliative care (SIPC) within the last decades [1–3]. In addition, patients’ self-assessment of multidimensional symptoms and problems (patient-reported outcomes: PROs) also grew in importance over the last years. Several studies have highlighted the relevance of incorporating patient perspectives alongside those of clinicians when developing palliative care measures [1–5]. A previous study demonstrated significant differences between patients’ and physicians’ ratings in specific symptoms even when using identical scales, underlining the importance of this double assessment and especially the need for obtaining self-assessments [6]. However, in SIPC daily clinical practice, systematic self-assessment often turns out to be complicated due to physical and cognitive impairments of these severely ill patients and can only be performed in minor proportion of patients [7, 8].

Treatment on a SIPC ward aims to improve physical as well as psychosocial and spiritual symptoms and problems of patients with advanced life-limiting diseases to later allow discharge to other palliative care services if needed and possible. These include specialist palliative home care teams, inpatient hospices, community hospice teams, day hospices and outpatient clinics [9]. Thus, multiprofessional treatment actually aims to focus, inter alia, on preparation for further care after discharge. However, in a varying proportion of up to 60-70% of patients [9] discharge will not be achievable and patients will later die on the SIPC ward. Understanding realistic aims of SIPC early after admission to the SIPC ward might help to focus treatment and communication with the patients and their family caregivers either on preparation for further care or on nearing death. Today only few socio-demographic or disease-related factors of prognostic impact are known, [10] and the prognostic impact of symptoms or specific constellations of physical and psychosocial problems is still unclear. Some previous studies aimed to define symptom clusters (SCs) that might help to understand the patients’ situation during palliative care more comprehensively [11–15]. A review analysis pointed out that these studies were indeed able to work out similar clusters grouping into four typical issues (anxiety/depression, nausea/vomiting, nausea/appetite loss, and fatigue/dyspnea/drowsiness/pain), but their impact and influencing factors remain inconsistently defined [16].

The underlying study aimed to evaluate patients’ main symptoms and problems at admission to a SIPC ward and their prognostic impact concerning dying on the ward. Symptoms were assessed in terms of self-assessment by patients and proxy-assessment by palliative care specialists (physicians). However the prognostic impact was analysed concerning the proxy-assessment only.

Secondary aims were (1) to analyse the prognostic value of symptoms when combined into symptom clusters (proxy-assessment only); and (2) to evaluate if proxy- and self-assessment of specific symptoms might differ or correspond.

## Methods

### Study design and participants

This prospective study evaluated patients’ self-assessment and proxy-assessment by palliative care specialists in a SIPC setting. They were obtained as routine assessments at the time of admission to the SIPC ward at the University Medical Center of Hamburg, Germany, between January 2016 and September 2018. Admission to the SIPC ward was caused by physical or psychosocial problems of the patients or their family caregivers prohibiting further patient care in an outpatient setting or on non-specialized hospital wards per definition. Proxy- and self-assessment was completed within 12 h of admission. All assessments were obtained weekly, however, the presented study focuses on the first assessment at patient’s admission. The study had been approved by the ethics committee of the Medical Association of Hamburg, Germany (PV4981).

### Measurements

#### Proxy-assessment reported by palliative care specialists

Palliative care specialists (physicians) completed the Symptom and Problem Checklist of the German Hospice and Palliative Care Evaluation, the HOPE-SP-CL, [17, 18] including the performance status according to the Eastern Co-operative Oncology Group (ECOG) [19, 20].

The HOPE-SP-CL actually consists of 16 items and one free entry (ratings from 0 to 3) with a maximum global sum score of 51. We adapted the questionnaire to 15 items rated from 0 (no) to 3 (severe) resulting in a HOPE-SP-CL Total Score from 0 to 45 with higher values representing high symptom burden [17, 18]. One item, wound care, was omitted due to the fact that it was only evaluated as present or not present in our routine assessment. One item, sleeping problems, was added, though not included in the Total Score. We also built three sub-scores: The HOPE-SP-CL Somatic Symptoms Score (pain, nausea, emesis, dyspnea, constipation, weakness, loss of appetite, tiredness) ranging from 0 to 24, the HOPE-SP-CL Psychological Symptoms Score (depression, tension, anxiety, confusion) ranging from 0 to 12, and the HOPE-SP-CL Social Care Score (social problems and nursing topics: assistance with activities of daily living (ADLs), overburden of family, problems with care planning) ranging from 0 to 9.

#### Self-assessment reported by patients

Patients’ self-assessment was performed using either the new version of the minimal documentation system for patients in palliative care (MIDOS), [21] which is the German version of the Edmonton Symptom Assessment Scale (ESAS), [22] or the Integrated Palliative Care Outcome Scale (IPOS) [23–27]. Additionally, all patients were asked to rate the Distress Thermometer of the National Comprehensive Cancer Center Network (NCCN) [28, 29].

The MIDOS was implemented between January 1st 2016 - August 15th 2016, and the IPOS between August 16th 2016 and September 30th 2018. We changed the assessment tool for daily practice, since the use of IPOS as a new tool was suggested by the European Association for Palliative Care (EAPC) and based on a publication with a first validation phase [25]. Also IPOS adds relevant new aspects (particularly psychosocial/spiritual aspects) as compared to the MIDOS.

The MIDOS originally contains 10 symptom items rated from 0 (no) to 3 (severe). These were included in the MIDOS Total Score, which ranges from 0 to 30 with higher values representing higher symptom burden. It also includes a question regarding the patients’ well-being, with ratings defined as “very bad”, “bad”, “moderate”, “good” and “very good” [21, 22]. Thirteen study-specific items were added to the MIDOS to complement practical, psychosocial and spiritual problems, each rated as the original MIDOS item. These items were adapted from the problem list which accompanies the DT and include problems with living situation, work/school, sorrows, loss of interest, sadness, nervousness, transportation, care of children, religious issues, loss of faith, insurances, and the relationship to their partner or relationship to their children [29]. Further tension and sleeping problems were added as they represent relevant symptoms in patients referred to SIPC.

The IPOS questionnaire consists of 17 items with ratings from 0 (most positive value = no symptom burden/problem) to 4 (most negative value = overwhelming symptom burden/problems). The IPOS Total Score ranges from 0 to 68 with higher values representing worse outcome [23–26]. Again, three sub-scores were calculated: the IPOS Physical Symptom Score (pain, shortness of breath, weakness or lack of energy, nausea, emesis, poor appetite, constipation, sore or dry mouth, drowsiness, poor mobility) ranges from 0 to 40, the IPOS Emotional Symptom Score (feeling anxious or worried about illness or treatment, family or friends being anxious or worried, feeling depressed, feeling at peace) from 0 to 16 and the IPOS Communication/Practical Score (Communication/practical issues: being able to share feelings with family and friends, having as much information as wanted, any practical problems being addressed) from 0 to 12. Another four items were added for comparability reasons, but none of them included in the Total Score: (1) patients’ well-being, as in the MIDOS, and (2) anxiety, (3) tension, (4) sleeping problems with ratings as in the IPOS.

The Distress Thermometer measures subjective distress within the previous week on an analogue scale rated from 0 “no distress” to 10 “extreme distress” [28, 29]. For the German version, a cut-off value of ≥5 reflects clinically relevant distress with need of professional psychological support [29].

### Statistical analyses

Descriptive statistics were calculated including frequency distributions, percentages, means, and standard deviations. For calculation of sum scores and sub-scores, 20% of missing values were tolerated. We imputed the missing values by the mean score for the missing items based on items completed by the individual. Most symptoms/problems assessed in this study were dichotomized: symptoms/problems were defined as being present whenever a level of at least moderate severity was reported.

Agreement between independent ratings of physicians (HOPE-SC-SL) and patients (MIDOS/IPOS) were examined using Cohen’s Kappa (ƙ), which is a chance-corrected measure of agreement (− 1 to 1). The comparison of physicians’ and patients’ dichotomized ratings was analysed for corresponding items only. We used the benchmarks by Landis and Koch, whereby 0.00 to 0.20, 0.21 to 0.40, 0.41 to 0.60, 0.61 to 0.80, and 0.81 to 1.00 indicate poor, fair, moderate, substantial, and almost perfect agreement, respectively [30].

Principal components analysis (varimax rotation with Kaiser normalization) was conducted to explore SCs based on physicians’ rating of the HOPE-SC-SL. Factor retention criteria were eigenvalue > 1 and the scree-plot method. The Kaiser-Meyer-Olkin (KMO) measure and Bartlett’s test of spherity were applied to examine appropriateness of factor analysis. A Cronbach’s α greater than or approximately 0.70 was considered as the criterion for an acceptable SC.

Multivariate binary logistic regression analyses were performed to assess the predictive value of the ECOG performance status (dichotomized) (1) with HOPE-SP-CL single symptoms, and (2) the HOPE-SP-CL SC including leading single symptoms (pain and dyspnea) for dying on the SIPC ward. Regarding SCs, new variables indicating the presence and absence of a SC for each patient were considered. The presence of at least 75% of the symptoms composing each SC was defined as requirement for its presence. Presence of single symptoms was determined with at least moderate symptom severity. Variables showing significance in bivariate regression analyses were selected for the multivariate regression models. Intercorrelation and multicollinearity were checked and did not pose a problem (spearman’s coefficient rho < 0.5, variance inflation factors < 1.4). All identified predictors were entered simultaneously into the regression analyses (method: enter) with adjustment for age. Missing data was handled by list-wise deletion and the strengths of associations were expressed as odds ratios (OR) with 95% confidence intervals (CI). All statistical analyses were performed using IBM SPSS 22 for Windows (IBM Corp., NY).

## Results

### Patient characteristics

A total of 1206 patients with a mean age of 68.0 years (SD 14.0 range, 20-97) were admitted to the SIPC ward between 01.01.2016-30.09.2018. About half of them were male and female (female *n* = 547, 45.4%; male *n* = 659, 54.6%). Most patients suffered from cancer, but 164 patients (13.6%) had non-malignant diseases. After a mean time of 10.2 days (SD 7.7; range, 1-64) staying on the SIPC ward, 251 patients (20.8%) were discharged to specialist palliative home care, 193 patients (16.0%) were admitted to inpatient hospice care and 712 (59.0%) died on the ward. Another 30 patients (2.5%) were admitted to nursing homes and 20 patients (1.7%) to other wards of the hospital for causal, mainly cancer treatment. Prior to admission, 656 patients (58.8%) had lived at home with family caregivers, 341 (30.6%) had lived at home alone, 103 (9.2%) in nursing homes, and 15 (1.3%) in other institutions. Additional details on patient characteristics are listed in Table 1.

**Table 1:** Patient characteristics (*N* = 1206)

		Pts.	%
Disease:	Lung cancer/thoracic cancer	188	15.6
	Pancreatic or hepatobiliary cancer	144	11.9
	Urogenital cancer	137	11.4
	Gynecologic cancer	111	9.2
	Hematological malignancies	89	7.4
	Cancer of the lower gastrointestinal tract	75	6.2
	Head and neck cancer	66	5.5
	Cancer of the neuro-cerebral system	65	5.4
	Cancer of the upper gastrointestinal tract	52	4.3
	Melanoma, dermatological cancer	33	2.7
	Sarcoma	34	2.8
	Other malignant diseases	48	4.0
	Cardiovascular diseases	52	4.3
	Neurological diseases	36	3.0
	Geriatric diseases, e.g. dementia	25	2.1
	Pneumological diseases	23	1.9
	Other non-malignant diseases	28	2.3
Admission to the SIPC ward from:	Oncology ward	308	25.5
	Emergency room	270	22.4
	Home care	215	17.8
	Internal medicine ward	84	7.0
	Intensive care unit	83	6.9
	Neurology/psychiatry ward	54	4.4
	Gynecologic ward	48	4.0
	Cardiologic ward	34	2.8
	Surgery ward	34	2.8
	Pneumologic ward	33	2.7
	Urologic ward	18	1.5
	Other wards	25	2.0
Advance directives:	Power of attorney (yes)	378	32.2
	Patient decree (yes)	343	29.3
	Legal guardianship (yes)	63	5.4
Treatment at admission:	Opioids (yes)	737	64.8
	Steroids (yes)	371	33.4
	Ongoing chemotherapy/oncologic treatment (yes)	93	8.5
	Ongoing radiotherapy (yes)	25	2.3

Pts, number of patients

SIPC, specialist inpatient palliative care

In the time while the MIDOS questionnaire was used for patients’ self-assessment, 271 patients (22.5% of the total cohort) were included, and another 935 patients (77.5% of the total cohort) while IPOS was used. Characteristics of both subgroups did not differ significantly (e.g. female 45% MIDOS/46% IPOS; mean age of 67.8 years (SD 12.3; range, 30-95)/65.3 years (SD 13.8; range, 20-95); non-malignant disease 3.6%/4.5%; admission from home care 23.2%/24.3%).

### Proxy-assessment reported by palliative care specialists

#### Symptom assessment

The HOPE-SP-CL assessment was completed by palliative care specialists (physicians) for 1184 patients (98.1% of the total cohort) at admission to the SIPC ward. The most frequent symptoms or problems for more than 50% of the patients of at least moderate intensity were weakness (95.3%), needs of assistance with activities of daily living (88.3%), organization of care (84.8%), overburdening of family caregivers (82.8%), tiredness (74.8%), appetite loss (74.4%), tension (66.2%), and pain (57.0%).

The mean HOPE-SP-CL Total Score was 24.6 of 45 (SD 5.9; range, 6-42), the HOPE-SP-CL Somatic Symptoms Score 12.0 of 24 (SD 3.7; range, 1-23), the HOPE-SP-CL Psychological Symptoms Score 5.6 of 12 (SD 2.5; range, 0-12), and the HOPE-SP-CL Social Care Score 7.0 of 9 (SD 1.8; range, 1-9). Patients presented with a mean ECOG performance status of 3.2 (SD 0.8; range, 1-4): 188 patients (16.6%) had an ECOG 0-2, 533 patients (47.1%) an ECOG 3 and 410 patients (36.3%) an ECOG 4.

#### Symptom clusters

Factor analysis of 15 HOPE-SP-CL items identified four clusters explaining 55.2% of the total variance: Deteriorated Physical Condition/Decompensation of Home Care (assistance with ADLs, weakness, tiredness, disorientation, organization of care, overburdening of family), Emotional (anxiety, tension, depression), Gastrointestinal (nausea, vomiting, appetite loss, constipation) and Other (dyspnea, pain). The Emotional cluster had Cronbach’s α > 0.80. The Deteriorated Physical Condition/Decompensation of Home Care cluster and the Gastrointestinal cluster had Cronbach’s α close to 0.70. The cluster of Other Symptoms consisted of clinically relevant symptoms, but did not show any internal consistency and could not be interpreted. Table 2 presents the results of the factor analysis.

**Table 2:** Symptom clusters identified by factor analysis (*n* = 955)

Symptom cluster based on HOPE-SC-SL	Symptoms	Factor analysis^a,b^				Cronbach’s α
		Deteriorated Physical Condition/Decompen-sation of Home Care	Emotional	Gastro-intestinal	Other	
**Deteriorated Physical Condition/Decompensation of Home Care**	Assistance with ADLs	.766	.041	-.051	.105	.682
	Weakness	.676	.071	.143	.289	
	Tiredness	.656	.036	.187	.148	
	Disorientation/confusion	.615	-.138	-.259	-.151	
	Organization of care	.555	.248	-.113	-.319	
	Overburdening of family caregivers	.464	.356	.029	-.285	
**Emotional**	Anxiety	.020	.835	.103	.095	.804
	Tension	.129	.824	.046	-.085	
	Depression	.030	.783	.155	.096	
**Gastrointestinal**	Nausea	-.139	.081	.857	-.078	.640
	Vomiting	-.097	-.030	.809	-.057	
	Appetite loss	.376	.149	.524	.245	
	Constipation	.120	.195	.497	-.128	
**Other**^**c**^	Dyspnea	.110	.218	-.060	.705	-.153
	Pain	-.023	.293	.277	-.427	

*ADLs* Activities of daily living

^a^Kaiser-Meyer-Olkin measure (KMO) = .740

^b^Bartlett’s test of sphericity, Approximate Chi-Square 3402.682, df(p) = 105(<.001)

^c^Not considered as a valid symptom cluster based on Cronbach’s α

The most common SC, Deteriorated Physical Condition/Decompensation of Home Care, was present in 950 (83.0%) patients. The Emotional and Gastrointestinal SCs were present in 342 (29.7%) and 204 patients (17.5%), respectively. Five hundred sixty-eight patients (51.3%) presented with one SC, 332 (30.0%) with two, and 69 (6.2%) with all SCs.

### Self-assessment reported by patients

#### Symptom assessment with MIDOS

Of 271 patients who were cared on the SIPC ward between 01.01.2016-10.08.2016, 57 had completed the MIDOS (21.0%). Reasons for not completing were weakness in 25 (9.2%) patients, cognitive impairment in 25 (9.2%), language barriers in 6 (2.2%) and patient refusal in another 6 patients (2.2%). In 152 (56.1%) cases no reason was documented. Whether help was needed for completing the questionnaire has not been recorded.

The mean MIDOS Total Score was 11.3 of 30 (SD 5.3; range, 3-23). Weakness (80.3%), tiredness (62.5%), appetite loss (60.4%), pain (50.9%), and sleeping problems (45.4%) were the five most frequently reported symptoms of at least moderate intensity. Patients rated their current overall well-being with 2.6 in mean (SD 1.0; range, 1-4) with “very bad” in 18.0%, “bad” in 20.0% and “moderate” in 48.0%. They stated problems with sorrows (63.6%), loss of interest (59.5%), sadness (56.5%), nervousness (37.2%), their living situation (36.7%), transportation (20.8%), care of children (16.3%), religious issues (12.2%), the relationship to their children (12.0%), insurances (6.4%), the relationship to their partner (6.1%), and loss of faith (2.6%). Problems with work or school were not addressed.

#### Symptom assessment with IPOS

The IPOS was completed by 314 of 935 patients (33.6%) who were hospitalized on the SIPC ward between 11.08.2016-30.09.2018. Patients completed it without help in 42.4%, with help of a family caregiver in 44.0% and with help of a health care professional in 4.8% (no information 8.8%). Patients were not able to complete the questionnaire due to severe weakness (19.0%), reduced overall condition (18.5%), cognitive impairment (13.7%), disorientation (2.9%), and language barriers (2.3%). Patients refused assessment in 2.3%, and in 41.3% assessment was not possible without information regarding the reason. The mean IPOS Total Score in 314 patients was 32.0 of 68 (SD 9.0; range, 9.7-61.0), the IPOS Physical Symptom Score 19.2 of 40 (SD 6.8; range, 3.0-35.6), the IPOS Emotional Symptom Score 9.4 of 16 (SD 3.1; 0.0-16.0), and the IPOS Communication/Practical Score 3.8 of 12 (SD 2.7; range, 0.0-11.0). Poor mobility (91.5%), weakness or lack of energy (91.2%), drowsiness (78.1%), poor appetite (67.9%), and pain (68.3%) were the five most frequently reported symptoms of at least moderate intensity. Further, patients rated their current overall well-being with 2.8 in mean (SD 0.84; range, 1-4) with “very bad” in 10.1%, “bad” in 18.9% and “moderate” in 57.1%. The three most frequently reported psychosocial problems were family or friends being anxious or worried about him/her (94.7%), feeling anxious or worried about his/her illness or treatment (85.7%), and feeling depressed (79.6%).

#### Assessment of subjective distress with distress thermometer

In the total cohort (*n* = 1206), 323 patients rated their distress with a mean intensity of 6.6 of 10 on the Distress Thermometer (SD 2.5; range, 0-10). Of these 263 (81.4%) indicated clinically relevant distress with need of professional psychological support (DT ≥ 5). In the “MIDOS group”, 48 patients scored their distress with 6.6 in mean (SD 2.5; range, 1-10), and 39 (81.3%) rated their distress with DT ≥ 5. Mean distress was 6.7 in 275 patients of the “IPOS group” (SD 2.5; range, 0-10) with DT ≥ 5 in 224 (81.5%).

### Agreement of symptoms as rated by physicians and - patients

The comparison of physicians’ and patients’ ratings was analysed for corresponding items as a secondary aim. Assessment with Kappa (ƙ) demonstrated moderate agreement between the independent symptom ratings of palliative care specialists (physicians) and patients related to pain (ƙ = 0.438) and dyspnea (ƙ = 0.503). The degree of agreement was fair in nausea, vomiting, constipation and appetite loss (ƙ = 0.297 to 0.394), and poor in anxiety, tension and sleeping problems (ƙ = 0.101 to 202). With regard to the remaining two symptoms of weakness and tiredness, ƙ was not significant. Table 3 demonstrates further details.

**Table 3:** Characteristics of physicians’ and patients’ symptom ratings and degree of rater agreement

Symptom	No. of assessments (paired N)	Characteristics of ratings by physicians (HOPE-SP-CL) and patients (MIDOS/IPOS)		Rater agreement by symptom/problem		
		Physicians n (%) present^a^	Patients n (%) present^a^	Percentage of actual agreement^b^	Kappa (ƙ)	Strength of agreement^c^
Pain	355	234 (65.9)	232 (65.4)	54.7	.438	Moderate
Nausea	348	106 (30.5)	113 (32.5)	73.9	.394	Fair
Vomiting	346	63 (18.2)	71 (20.5)	78.1	.297	Fair
Dyspnea	351	143 (40.7)	141 (40.2)	76.1	.503	Moderate
Constipation	342	152 (44.4)	151 (44.2)	62.8	.248	Fair
Weakness	351	330 (94.0)	315 (89.7)	84.3	-.009	Not significant
Appetite loss	350	269 (76.9)	236 (67.4)	72.3	.318	Fair
Tiredness	349	239 (68.5)	264 (75.6)	63.1	.088	Not significant
Anxiety^d^	348	179 (51.4)	142 (40.8)	56.0	.125	Poor
Tension^e^	338	231 (68.3)	166 (49.1)	54.7	.101	Poor
Sleeping problems^f^	346	132 (38.2)	194 (56.1)	59.0	.202	Poor

HOPE-SP-CL, Symptom and Problem Checklist of the German Hospice and Palliative Care Evaluation

MIDOS, New version of the minimal documentation system for patients in palliative care

IPOS, Integrated Palliative Care Outcome Scale

^a^dichotomized symptoms: “not present”= no/mild (HOPE-SP-CL) or none/little (MIDOS/IPOS); “present”= moderate/severe (HOPE-SP-CL) or moderate/severe/overwhelming (MIDOS/IPOS)

^b^Percentage of ratings matched (both ratings either yes or no)

^c^As per Landis and Koch 1977

^d^study-specific item added to IPOS

^e^study-specific item added to MIDOS and IPOS

^f^study-specific item added to MIDOS, IPOS and HOPE-SP-CL

### Prognostic impact on outcome

Multivariate regression analysis based on HOPE-SP-CL single symptoms revealed that, when adjusting for patient’s age, dying on the SIPC ward was predicted by ECOG performance status of 3/4 (OR 2.355) as well as dyspnea (OR 2.381), appetite loss (OR 1.421), tiredness (OR 1.946), and disorientation/confusion (OR 1.787) with at least moderate intensity. The model explained 23% of the total variance (Nagelkerke’s R^2^: .232). Details are presented in Table 4.

**Table 4:** Multivariate logistic regression analysis for dying on the SIPC ward

	Results of bivariate logistic regression for dying on the SIPC ward				Results of multivariate logistic regression for dying on the SIPC ward			
	β	SE	OR (95% CI)	p	β	SE	OR (95% CI)	p
Age	.026	.004	1.026 (1.017, 1.035)	**<.001**	.015	.006	1.015 (1.005, 1.026)	.005
**Performance status**								
ECOG 3/4	1.451	.174	4.269 (3.035, 6.005)	**<.001**	.856	.245	2.355 (1.457, 3.805)	**<.001**
**Symptom severity**								
Pain (moderate/severe)	.118	.120	1.125 (.889, 1.423)	.327	^a^			
Nausea (moderate/severe)	-.297	.141	.743 (.564, .980)	**.035**	-.017	.212	.983 (.649, 1.489)	.937
Vomiting (moderate/severe)	-.377	.176	.686 (4.486, .967)	**.032**	-.409	.257	.664 (.401, 1.099)	.111
Dyspnea (moderate/severe)	.995	.125	2.706 (2.120, 3.454)	**<.001**	.868	.149	2.381 (1.777, 3.191)	**<.001**
Constipation (moderate/severe)	.060	.123	1.062 (.835, 1.350)	.626	^a^			
Weakness (moderate/severe)	1.031	.292	2.803 (1.583, 4.964)	**<.001**	.270	.393	1.310 (.606, 2.829)	.492
Appetite loss (moderate/severe)	.600	.136	1.822 (1.396, 2.378)	**<.001**	.351	.173	1.421 (1.012, 1.995)	**.043**
Tiredness (moderate/severe)	.947	.139	2.578 (1.963, 3.385)	**<.001**	.666	.170	1.946 (1.395, 2.714)	**<.001**
Wound care (yes)	.133	.158	1142 (.837, 1.557)	.402	^a^			
Assistance with ADLs (moderate/severe)	1.516	.203	4.554 (3.057, 6.782)	**<.001**	.223	.282	1.249 (.719, 2.173)	.430
Feeling depressed (moderate/severe)	-.063	.121	.939 (.741, 1.190)	.605	^a^			
Anxiety (moderate/severe)	.158	.119	1.171 (.927, 1.479)	.186	^a^			
Tension (moderate/severe)	.140	.126	1.151 (.899, 1.473)	.265	^a^			
Disorientation/confusion (moderate/severe)	.965	.134	2.624 (2.019, 3.411)	**<.001**	.581	.167	1.787 (1.289, 2.478)	**<.001**
Organization of care (moderate/severe)	.625	.164	1.868 (1.353, 2.578)	**<.001**	.202	.221	1.224 (.794, 1.887)	.359
Overburdening of family (moderate/severe)	.469	.157	1.599 (1.175, 2.176)	**.003**	.231	.214	1.259 (.828, 1.915)	.281

Reference group: Patient’s discharge from palliative care ward (binary logistic regression analyses)

Reference values: ECOG: 0-2; HOPE-SP-CL: wound care: no, remaining symptoms: none/mild

Multivariate regression analysis: *N* = 958 patients; tolerance values between .642 und .959; Nagelkerke’s R^2^: .232

ECOG, Performance Status according to the Eastern Co-operative Oncology Group

ADLs, Activities of daily living

HOPE-SP-CL, Symptom and Problem Checklist of the German Hospice and Palliative Care Evaluation

SIPC, specialist inpatient palliative care

^a^not included in multivariate regression analysis due to result of bivariate regression analysis

Multivariate regression analysis based on SC evaluation showed that, when adjusting for patient’s age, ECOG performance status of 3/4 (OR 2.679), presence of the cluster Deteriorated Physical Condition/Decompensation of Home Care (OR 2.372) and presence of dyspnea (OR 2.448) predicted dying on the SIPC ward (all *p* < 0.001). The model explained 19% of the total variance (Nagelkerke’s R^2^: .185). For details see Table 5.

**Table 5:** Multivariate logistic regression model for dying on the SIPC ward according to symptom clusters

	Results of bivariate logistic regression for dying on the SIPC ward				Results of multivariate logistic regression for dying on the SIPC ward			
	β	SE	OR (95% CI)	p	β	SE	OR (95% CI)	p
Age	.025	.004	1.025 (1.017, 1.034)	**<.001**	.019	.005	1.019 (1.009, 1.029)	**<.001**
**Performance status**								
ECOG 3/4	1.451	.174	4.269 (3.035, 6.005)	**<.001**	.992	.196	2.679 (1.836, 3.961)	**<.001**
**Presence of HOPE-SP-CL symptom cluster**								
Deteriorated Physical Condition/Decompensation of Home Care	1.287	.166	3.621 (2.614, 5.017)	**<.001**	.864	.190	2.372 (1.636, 3.440)	**<.001**
Gastrointestinal	-.257	.155	.774 (.571, 1.048)	.098	^a^			
Emotional	.084	.131	1.088 (.841, 1.407)	.521	^a^			
**Presence of leading symptoms**								
Pain (moderate/severe)	.118	.120	1.125 (.889, 1.423)	.327	^a^			
Dyspnea (moderate/severe)	.995	.125	2.706 (2.120, 3.454)	**<.001**	.895	.135	2.448 (1.877, 3.192)	**<.001**

Reference group: Patient’s discharge from palliative care ward (binary logistic regression analyses)

Reference values: ECOG: 0–2; HOPE-SP-CL symptom clusters: absence of symptom cluster; single symptoms: no/little pain and dyspnea

Multivariate regression analysis: *N* = 1082 patients; Nagelkerke’s R^2^ = .185

ECOG, Performance Status according to the Eastern Co-operative Oncology Group

HOPE-SP-CL, Symptom and Problem Checklist of the German Hospice and Palliative Care Evaluation

SIPC, specialist inpatient palliative care

^a^not included in multivariate regression model due to result of bivariate analysis

## Discussion

This prospective study analysed systematic assessment of symptoms and problems (self- and proxy-assessment) in a large cohort of more than 1200 patients immediately after admission to an SIPC ward and the prognostic impact of the proxy-assessment results. Additionally, the prognostic value of symptoms when combined into symptom clusters (proxy-assessment) was evaluated as well as agreement between proxy- and self-assessment.

Both, proxy- and self-assessment, revealed weakness, impaired mobility need of assistance with activities of daily living, overburdening of family caregivers, and tiredness as the most frequent symptoms or problems with at least moderate intensity in minimum 70% of patients. Probably, these problems and symptoms have caused the admission to the SIPC ward. Additional symptoms were appetite loss and pain. This is consistent with previous studies using HOPE-SP-CL assessment on German SIPC wards, in which most frequent symptoms of at least moderate intensity were in descending order: weakness, need of assistance with activities of daily living, tiredness, appetite loss, and overburdening of family caregivers with 91-65% [11, 17]. Overall, problems and symptoms impairing autonomy and mobility, but not primarily pain or dyspnea, seem to be the main reasons for admission to SIPC wards at least in Germany. In contrast, other studies reported uncontrolled pain as most prevalent reasons for admission to SIPC wards in cancer patients [31]. This heterogeneity might be caused by different admission policies in different countries, the focus on cancer patients only, or the use of different assessment instruments. Patients in need for early-integrated specialist palliative care during cancer treatment, or those requesting additional specialist palliative care support, mostly suffer from tiredness, weakness, pain, appetite loss and dyspnea in at least moderate intensity [32]. This might demonstrate that pain and dyspnea are even more relevant in early advanced cancer disease, while impairment of mobility and autonomy gain in relevance later on [33]. With a mean ECOG performance status of 3.2 (of a maximum of 4) and more than half of the patients dying on the SIPC ward, our cohort includes a majority of patients in a late stage disease. The vulnerability of our patients is also evident from the fact that about 40% of our patients were admitted via the emergency room or directly from home care, which suggests that a SIPC treatment was not proactively planned or not sufficient, but an emergency admission necessary. Other studies with potentially other admission policies report considerably lower ECOG performance status in oncology patients on a palliative care unit of ECOG 2 in median [34].

In contrast to MIDOS, IPOS assessment includes additional psychosocial problems with family or friends being anxious or worried about him/her, feeling anxious or worried about his/her illness or treatment, and feeling depressed. 80-90% of patients reported these aspects as being present with at least moderate intensity. This high impact of psychosocial aspects is also reflected by the IPOS Total Score (32.0 of 68: 47%), which might indicate a higher burden compared to MIDOS Total Score (11.3 of 30: 38%), which mainly asks for physical problems. Patients’ high psychosocial burden in their last weeks and days of life got also evident in the high mean distress level, which indicates that about 80% of 323 patients were in need of professional psychological support.

Conduct of self-assessment could be improved over time by repeated training of all health care professionals on the SIPC ward. During the first months of assessment, and the use of MIDOS, 21% of 271 patients completed the questionnaire. After changing the self-assessment tool to IPOS 34% of 935 patients completed the questionnaire. The increase of completed IPOS compared to MIDOS is probably due to restructuring with implementing the IPOS (e.g. reminder routines and addressing family caregivers to help) as also shown in previous studies [25, 27]. However, about half of these patients needed additional help by family caregivers or healthcare professionals, which we can conclude from the IPOS data assessed only, since this information was not obtained in the MIDOS. Several previous studies have also experienced difficulties of self-assessment in palliative care settings with rates of completed questionnaires ranging from 22 to 53% with increased rates after staff training [7, 34, 35]. Overall, comprehensive self-assessment seems to be limited to a minor proportion of patients during SIPC. Therefore, further knowledge about specific impact of self-assessments might help to adapt the self-assessment to patients’ individual possibilities.

In our study, palliative care specialists’ assessment showed correlation with the patients’ self-assessment concerning some corresponding items. Agreement between patients and palliative care specialists was highest for “classical physical symptoms” as pain and dyspnea followed by nausea, vomiting, constipation and appetite loss. In contrast, for anxiety, tension and sleeping problems, weakness and tiredness, which represent symptoms with high relation to psychosocial and care-related aspects, agreement was poor or missing. These results strengthen previous data (same setting) demonstrating significant differences in ratings between patients and palliative care physicians with regard to tiredness, lack of energy, anxiety, and sadness, which were mainly underestimated by the physicians [6]. Therefore, routine assessment should focus especially on psychosocial- and care-related aspects in those patients in whom a complete assessment is not possible due to physical impairment. However, patients in palliative care settings often suffer from cognitive impairment, which impedes a self-assessment but also renders proxy-assessment difficult. Nevertheless it is recommended, since it is the only possible approach to assess symptom burden [17, 36]. We ensured that experienced palliative care specialists completed the assessment.

Multivariate regression analysis confirmed, as anticipated, higher ECOG performance status as a significant predictor for dying on the SIPC ward, but also symptoms aside from the performance status like at least moderate dyspnea, appetite loss, tiredness, and disorientation/confusion as significant predictors. A previous study showed higher scores for fatigue, drowsiness, shortness of breath, and appetite loss to be associated with dying on the SIPC ward [10]. Future studies should prospectively evaluate the prognostic impact of specific symptom complexes, like dyspnea/shortness of breath, tiredness/fatigue, and disorientation/confusion/drowsiness during SIPC.

In our study four SC of the HOPE-SP-CL assessment could be identified: (1) Deteriorated Physical condition/Decompensation of Home Care, (2) Emotional, (3) Gastrointestinal and (4) Other, though the latter could not be interpreted due to low internal consistency. Thus our results were not completely similar to previously described SCs: (1) Anxiety/Depression, (2) Nausea/Vomiting, (3) Nausea/Appetite loss, and (4) Fatigue/Dyspnea/Drowsiness/Pain [16]. Another previous study also using HOPE-SP-CL assessment reported five SCs: (1) Nausea/Vomiting; (2) Anxiety/Tension/Feeling Depressed; (3) Wound Care/Disorientation/Confusion; (4) Organization of Care/Overburdening of Family; (5) Weakness/Tiredness/Need for Assistance with Activities of Daily Living/Appetite Loss. The approach to distinguish between cancer and non-cancer patients was not possible by SCs [11]. However, predictive impact of SC analyses was observed in two other studies using other assessment tools. The presence of two or more SCs had prognostic relation to survival in a Portuguese Tertiary Palliative Care Clinic [13]. Another study demonstrated a negative impact of four SCs occurring during palliative chemotherapy on functioning and quality of life in advanced cancer patients [14]. In contrast, in our study, only one broad SC, Deteriorated Physical Condition/Decompensation of Home Care, showed prognostic impact concerning later dying on the SIPC ward versus discharge.

This study is characterized by its strengths, including the large patient number and evaluation of routine assessment, but has also some notable limitations. First, it represents a single center experience, which might not be completely transferable to all SIPC wards. In addition two different questionnaires were used, which impedes comparability. Also we know that almost 50% of patients using the IPOS had help completing it, whereas there is no data for the MIDOS available. We cannot determine the influence of informal or professional caregivers support concerning the answers.

Evaluation of specific psychological distress, such as levels of anxiety and depressive symptoms, is limited, since we only used the MIDOS/IPOS and the Distress Thermometer but no specialized psychological tool. Additionally physician - patient agreement was only analysable for corresponding symptoms in the assessments used, which narrows the scope of its significance.

## Conclusions

In conclusion, problems and symptoms impairing autonomy and mobility, including weakness, tiredness or needing assistance with daily living activities, but not primarily pain or dyspnea, lead to acute admissions to SIPC wards. Our study indicates that comprehensive patient self-assessment can be improved by staff training, but remains limited to a minor proportion of patients during SIPC. Palliative care specialists’ assessment is almost similar to the patients’ concerning physical symptoms, especially pain, dyspnea, nausea, vomiting, constipation and appetite loss, while their estimation of psychosocial and care-specific aspects seems rather insufficient. Therefore, routine assessment should particularly focus on psychosocial and care-related aspects in those patients in whom a complete self-assessment of physical and psychosocial problems is not possible. Self-assessment with Distress Thermometer and IPOS adds important information on the patients’ psychosocial situation – the first as a screening, the second with detailed information. Higher symptom severity in dyspnea, appetite loss, tiredness, and disorientation/confusion as well as an increased ECOG as assessed at admission, seem to be predictive for higher probability of dying on the SIPC ward.

## Data Availability

All data analysed during this study are included in this published article.

## Abbreviations

ADLs: Activities of daily living
CI: Confidence interval
DT: Distress Thermometer
ESAS: Edmonton Symptom Assessment Scale
HOPE-SP-CL: Symptom and Problem Checklist of the German Hospice and Palliative Care Evaluation
IPOS: Integrated Palliative Care Outcome Scale
MIDOS: New version of the minimal documentation system for patients in palliative care
NCCN: National Comprehensive Cancer Center Network
OR: Odds ratio
PRO: Patient-reported outcome
SC: Symptom cluster(s)
SIPC: Specialist inpatient palliative care
